# Supplementary material for: Industrialization drives the gut microbiome and resistome of the Chinese populations
Source: mSystems. 2025 Feb 4;10(3):e01372-24. doi: 10.1128/msystems.01372-24 (PMC11915869; doi:10.1128/msystems.01372-24)
Supplement: Supplemental Figures — Fig. S1 to S6. [file msystems.01372-24-s0001.pdf]

Supplementary Figure 1

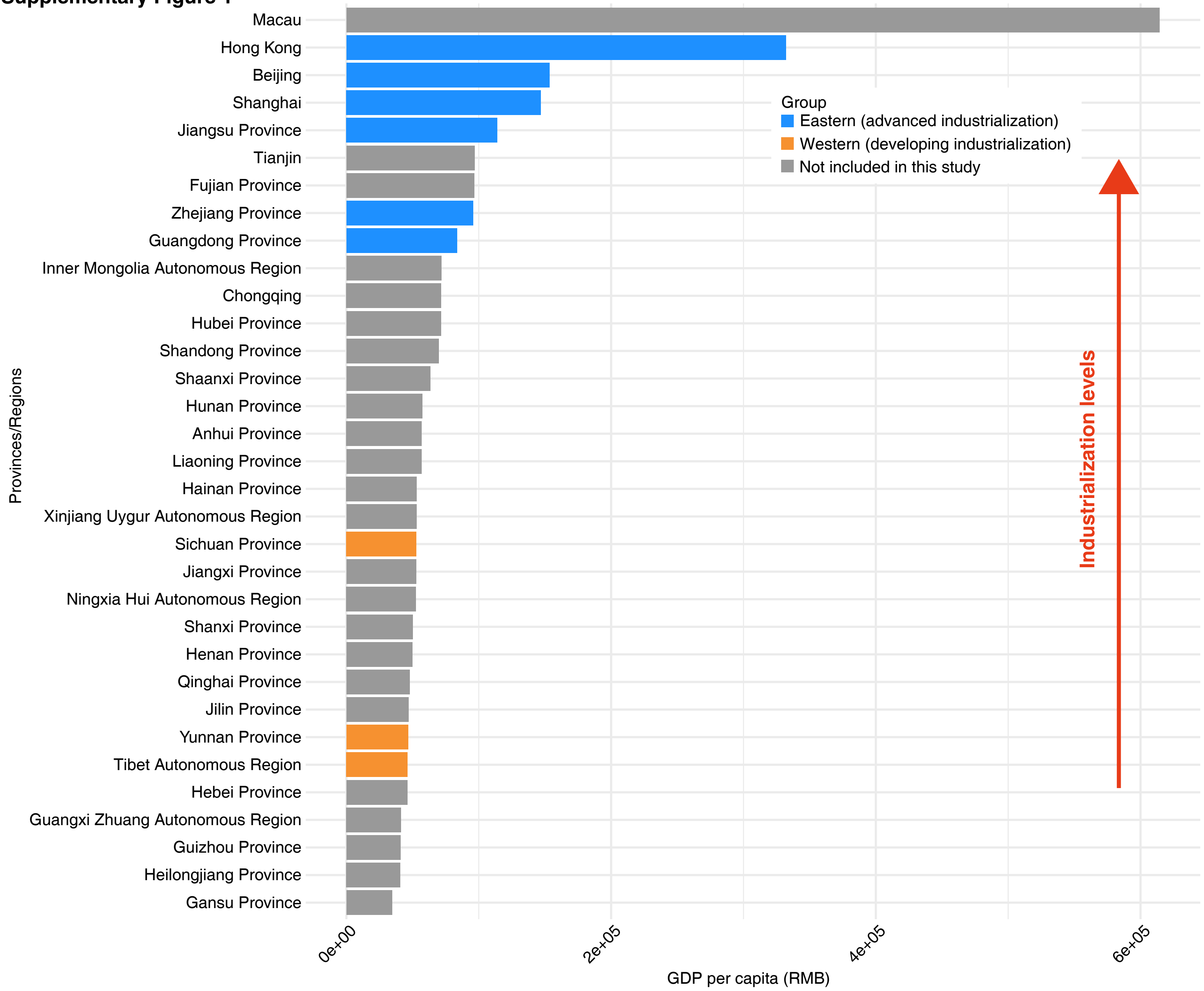

Supplementary Figure 2

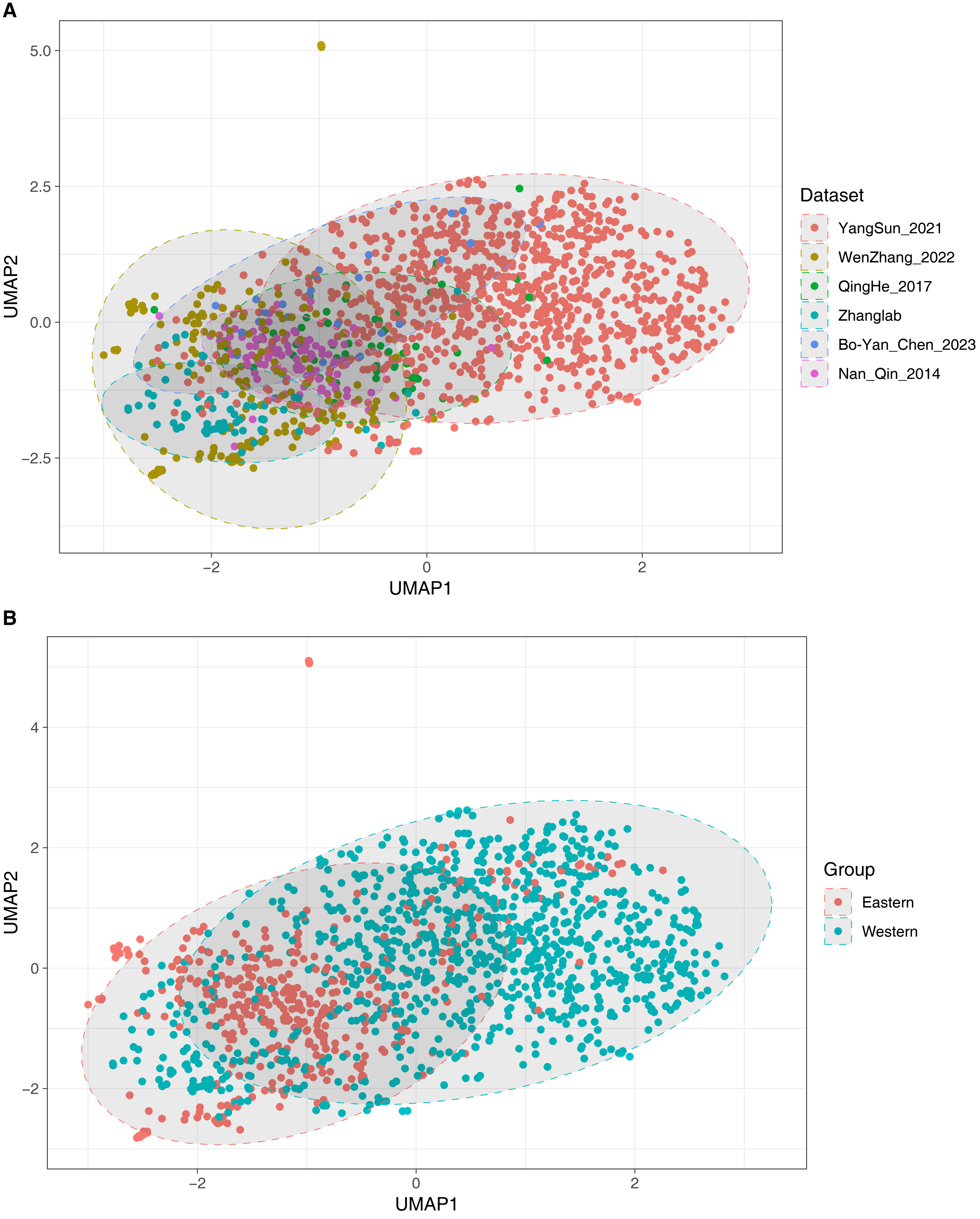

Supplementary Figure 3

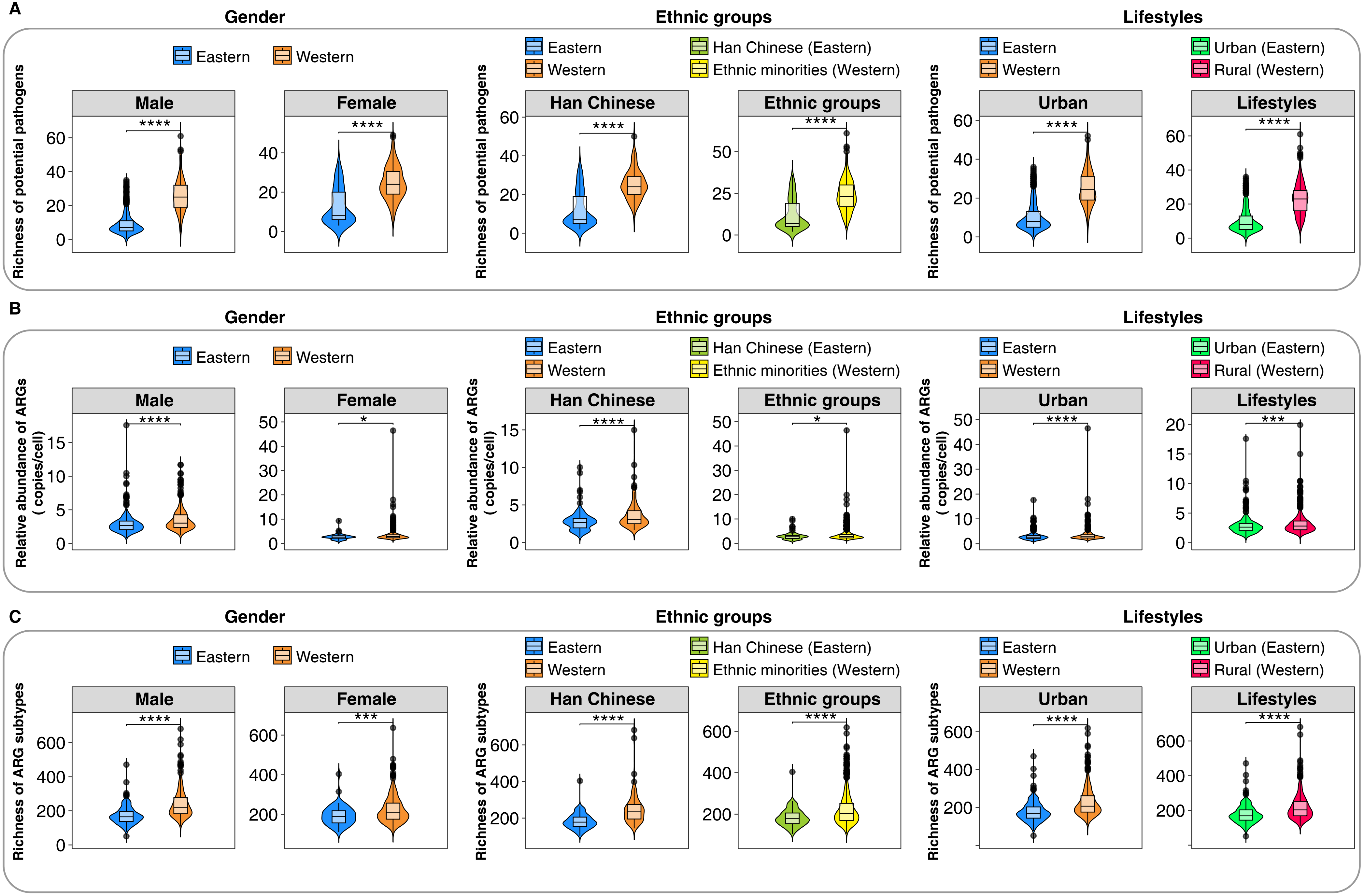

Supplementary Figure 4

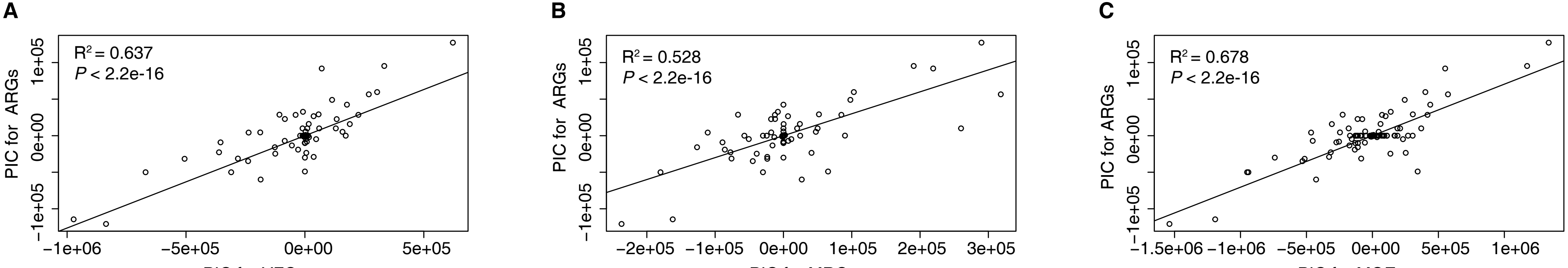

Supplementary Figure 5

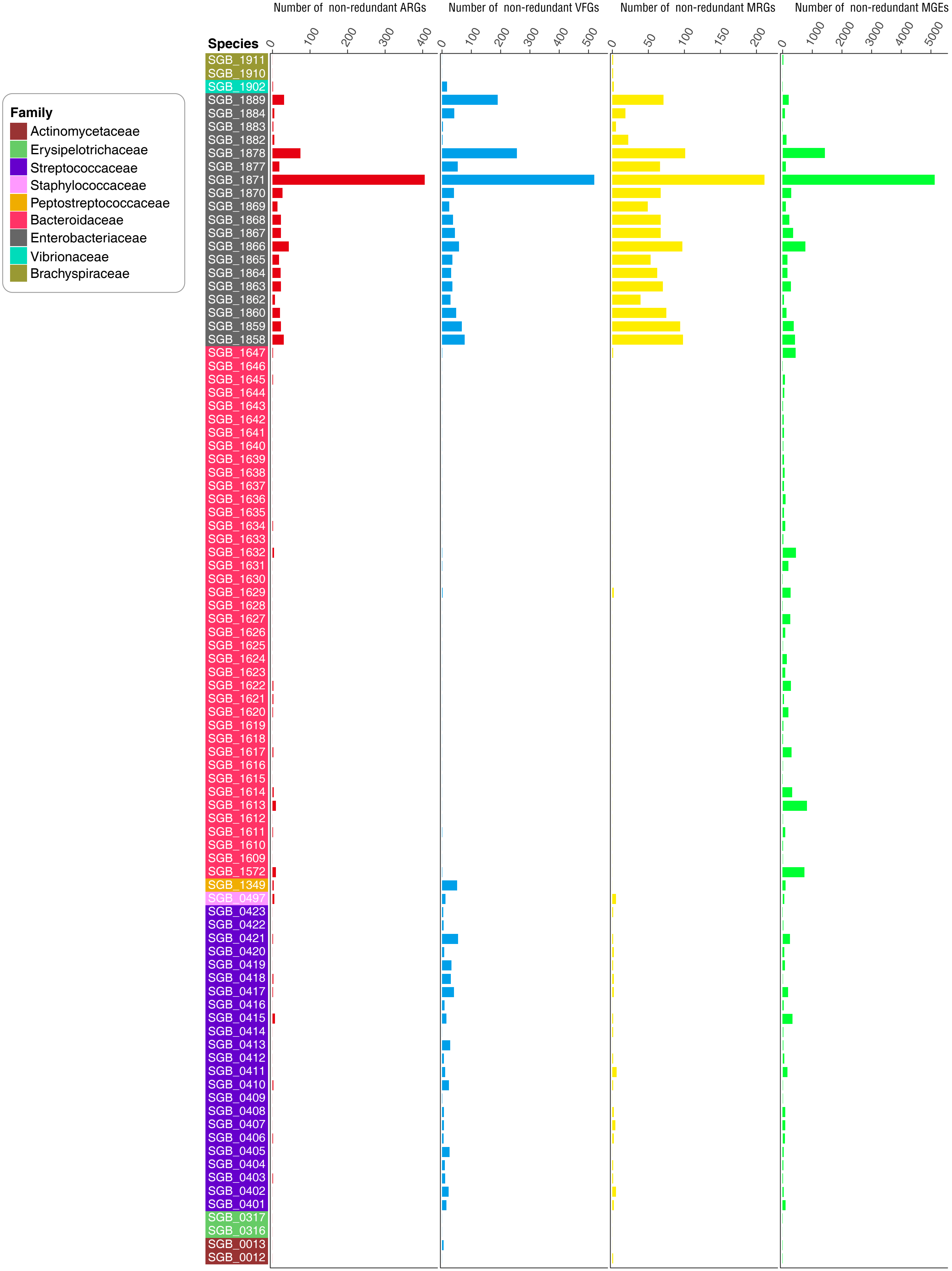

Supplementary Figure 6

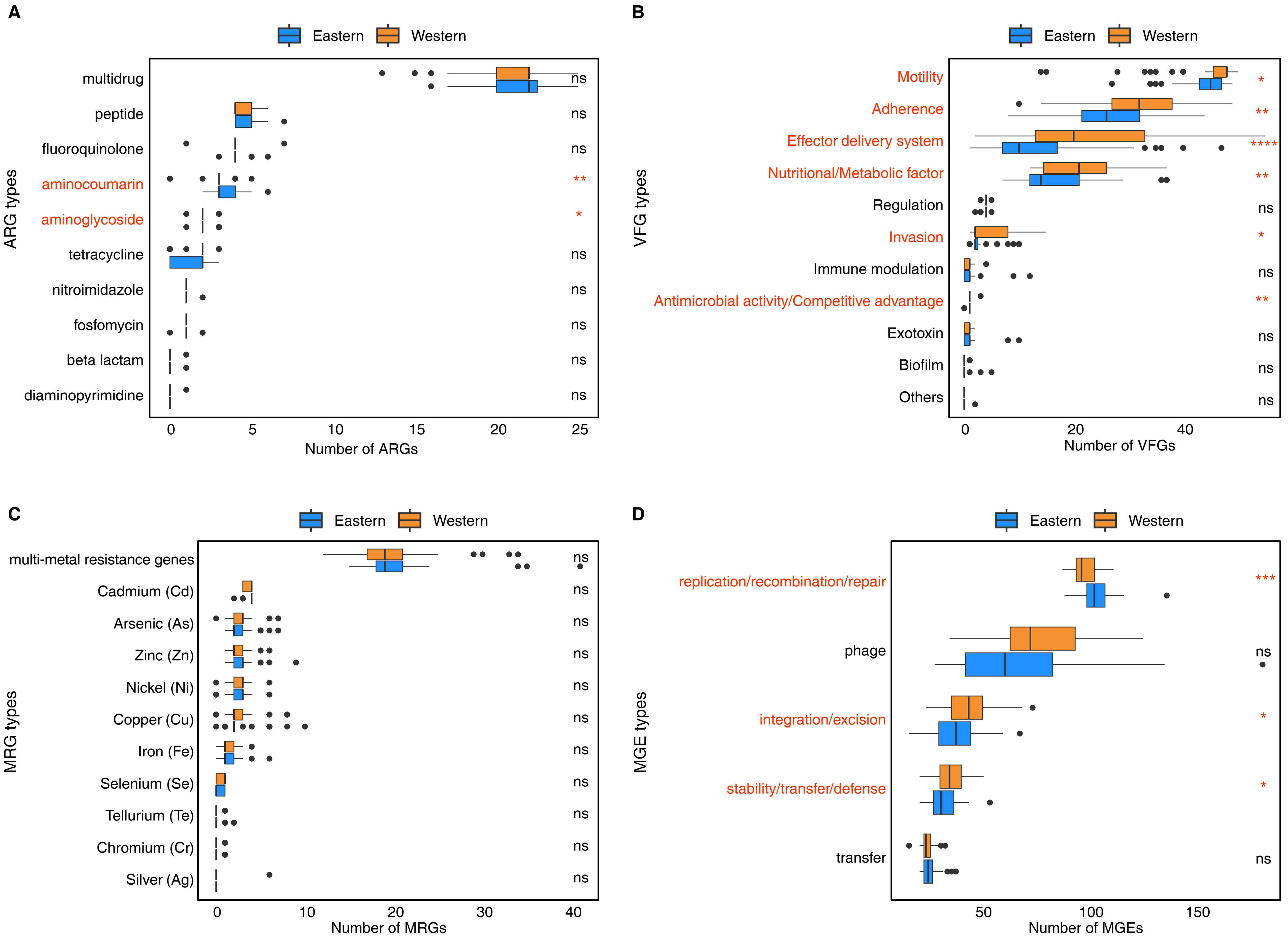

Supplementary Figure Legends

Supplementary Figure 1. Industrialization is a process characterized by the sustained increase in per capita income. According to the data from the National Bureau of Statistics, the bar chart shows the GDP per capita of various provinces and regions in China over the past decade. In this study, the eastern region is defined as having advanced industrialization, ranking among the top 10 in the country, while the western region is characterized by developing industrialization due to its relatively lower GDP per capita (Methods).

Supplementary Figure 2. UMAP of taxonomic profiles based on Euclidean distance between samples. (A) UMAP of taxonomic profiles colored by datasets. Zhanglab is a dataset for the newly sequenced data in this study. **(B)** UMAP of taxonomic profiles colored by groupings in this study. Subclusters identified by UMAP of taxonomic profiles roughly corresponded to groupings in this study but not datasets.

Supplementary Figure 3. Comparative richness of potential pathogens, relative abundance of ARGs, and richness of ARG subtypes between Eastern and Western populations. (A) Comparison of the richness of potential pathogens between eastern and western populations, stratified by gender, ethnic groups, and lifestyles. **(B)** Comparison of the relative abundance of ARGs between eastern and western populations, stratified by gender, ethnic groups, and lifestyles. **(C)** Comparison of the richness of ARG subtypes between eastern and western populations, stratified by gender, ethnic groups, and lifestyles. The Han and minority ethnic groups in the western populations were compared separately with the Han ethnic group in the eastern populations. The urban and rural residents in the western populations were compared separately with the urban residents in the eastern populations. The chosen significance level for the statistical test employed Wilcoxon rank sum test, *$P < 0.05$, ***$P < 0.001$, ****$P < 0.0001$.

Supplementary Figure 4. Phylogenetically Independent Contrasts (PICs) analysis was conducted to investigate the relationships between ARGs and VFGs, ARGs and MRGs, and ARGs and MGEs in the genomes of potential pathogens.

Supplementary Figure 5. Statistics of the number of non-redundant ARGs, VFGs,

MRGs, and MGEs in the genomes of 91 potentially pathogenic microbial species assembled in this study.

Supplementary Figure 6. Comparison of the types of ARGs, VFGs, MRGs and MGEs carried by *E. coli* in the gut microbiota between eastern and western populations. **(A)** Comparison of the number of different types of ARGs carried by Escherichia coli in the gut microbiota between eastern and western populations. **(B)** Comparison of the number of different types of VFGs carried by Escherichia coli in the gut microbiota between eastern and western populations. **(C)** Comparison of the number of different types of MRGs carried by Escherichia coli in the gut microbiota between eastern and western populations. **(D)** Comparison of the number of different types of MGEs carried by Escherichia coli in the gut microbiota between eastern and western populations. The chosen significance level for the statistical test employed Wilcoxon rank sum test, *$P < 0.05$, ** $P < 0.01$, ***$P < 0.001$, ****$P < 0.0001$.
